# Ethanol inactivation of orthonairoviruses in ixodid ticks

**DOI:** 10.1007/s10493-021-00656-w

**Published:** 2021-09-20

**Authors:** A. Schulz, K. Methling, M. Lalk, A. Eisenbarth, M. Keller, M. H. Groschup

**Affiliations:** 1 Friedrich-Loeffler-Institut, Institute of Novel and Emerging Infectious Diseases, Südufer 10, 17493 Greifswald-Insel Riems, Germany; 2 Institute of Biochemistry, University of Greifswald, Felix-Hausdorff-Straße 4, 17489 Greifswald, Germany; 3 Bundeswehrkrankenhaus Hamburg, Abt. XXI, Außenstelle BNITM, Bernhard-Nocht-Str. 74, 20359 Hamburg, Germany

**Keywords:** Ticks, Inactivation, Ethanol, Tick-borne pathogens, Quantitative ^1^H-NMR spectroscopy

## Abstract

Ixodid ticks represent vectors and reservoirs for a broad range of zoonotic pathogens. Collected ticks from field studies are therefore usually stored in ethanol, which in higher concentrations effectively inactivates most of the known tick-borne pathogens. Although commonly practiced as gold standard for inactivation, hardly any scientific data demonstrate that ethanol sufficiently penetrates the comparatively thick cuticula of ticks. Therefore, *Amblyomma hebraeum* tick pools were stored for 21 days in ethanol (96%). Afterwards, the ethanol was removed and the ticks were homogenized. Quantitative ^1^H-NMR spectroscopic analysis was applied to determine the residual concentration of ethanol inside the ticks. ^1^H-NMR spectroscopic analysis revealed that ethanol constituted 28.3–42.6 mg of the total weight of three ticks in the pools (89.9–121.5 mg). In addition, the low-pathogenic Hazara orthonairovirus (HAZV) was used as a cell culture model for this study. The virus was exposed to ethanol concentrations between 0 and 60% and incubated under various temperature conditions for four time periods. Afterwards, the residual virus infectivity was determined by titration. Following ethanol exposure, HAZV did not grow in cells after 9 h of exposure to an ethanol concentration of 25%. These results demonstrate an extremely low ethanol resistance of the virus, which was generally in line with previously reported ethanol inactivation data for Crimean-Congo hemorrhagic fever orthonairovirus (CCHFV). After prolonged storage and impregnation, comparable ethanol concentrations are achieved in the ticks, indicating the suitability of this inactivation method also for Bunyaviruses in ticks. At the very least, a massive virus inactivation can be assumed. Definitive proof of virus inactivation would require a bioassay of ethanol-treated infected ticks under appropriate biosafety conditions.

## Introduction

Ixodid ticks are vectors and reservoirs for a broad variety of highly pathogenic zoonotic bacteria and viruses potentially causing life-threatening diseases (bacteria: Anaplasmosis, Babesiosis, Borreliosis, etc.; viruses: Tick borne Encephalitis, Omsk hemorrhagic fever, Crimean-Congo hemorrhagic fever, Severe Febrile Thrombocytopenia Syndrome, etc. (for a more extensive list, see https://www.cdc.gov/ticks/diseases/index.html). One of the most grievous tick-borne viruses, classified as biosafety level 4 pathogen, is Crimean-Congo hemorrhagic fever orthonairovirus (CCHFV). This virus belongs to the *Nairovididae* family in the *Orthonairovirus* genus and is transmitted by ticks of the genus *Hyalomma*, being its main vector and reservoir (Bente et al. 2013; Gargili et al. 2017). Humans can develop severe disease symptoms including fever, nausea and hemorrhagic bleedings with lethality rates of up to 30% (Thangamani and Bente 2014; Whitehouse 2004). Studying and diagnostic screening of ixodid ticks and their zoonotic pathogens has therefore become an essential part of modern public health research. However, in order to safely handle and taxonomically identify tick species possibly infected with CCHFV at a lower biosafety level, the virus must therefore be reliably inactivated.

For this purpose and for their preservation, ticks are usually stored in ethanol, which at higher concentrations effectively inactivates most of the known tick-borne pathogens over time. While the inactivation kinetics for individual pathogens can be deduced from in vitro cell culture studies (Kampf 2018), there are hardly any, if at all, scientific data available on the actual rate of penetration of ethanol into the tissue of ticks. Diffusion of the small polar ethanol molecules is likely, but it may also be hindered by the strong chitin cuticle ixodid ticks possess, which is composed of a polysaccharide matrix of N-acetylglucosamine units, crosslinked together with proteins, and of lipids. Depending on the protein types and the density and thickness of chitin, the cuticular exoskeleton can be extremely durable and solid. However, this hull is penetrated by orifices such as the mouth stomata, the spiracles and the genital as well as anal pores. Furthermore, the cuticle shields are, as common with all arthropods, connected and therefore interrupted by junctions and joints. Hence, it is plausible that the ethanol penetrates preferentially along these routes, but data on ethanol concentrations inside ethanol immersed ticks have never been determined properly.

Therefore, the main objective of the study was to determine the ethanol concentration in ticks after a defined submersion time in 96% alcohol. As this study was primarily motivated in order to facilitate working with CCHFV, we screened the literature for reports on the alcohol resistance of this virus. A previous study showed that CCHFV was already undetectable after 2 min in 30% ethanol using an in vitro cell culture system (Hardestam et al. 2007). We therefore determined the ethanol stability of Hazara orthonairovirus (HAZV), which is a very closely related but non-pathogenic relative of CCHFV, in a similar cell culture system. The goal of the study was to prove that (1) ethanol does indeed diffuse into the tick body and (2) thereby accumulates in internal concentrations suitable for virus inactivation.

## Materials and methods

### Measurement of ethanol in ixodid ticks

In the absence of a *Hyalomma* tick colony, *Ambylomma (A.) hebraeum* ticks, originating from a laboratory colony (kindly provided by MSD Animal Health Innovation, Schwabenheim, Germany) were used as another ixodid tick species for evaluating the permeability of ethanol into a tick corpus. This tropical hard tick is well suited as a model, as it is a very large species similar to most *Hyalomma* spp. ticks.

Seven tick pools were stored for 21 days at room temperature in 1.5 ml absolute ethanol (96%) using 2-ml Eppendorf tubes. Each tick pool consisted of three adult *A. hebraeum* specimens (unfed) of the same sex. Overall, four pools of males (12 ticks in total) and three pools of females (nine ticks in total) were examined. After 21 days of exposure, the ticks were removed from the ethanol and rinsed with distilled water. Exceeding water was wiped off with absorbent paper towel. Subsequently, the tick pools were homogenized in 1 ml of distilled water using a 5-mm-diameter steel bead. The weight of the samples was determined by precision balance at all crucial stages of the procedure. After centrifugation (10,000 rpm; 20 min), 500 µl supernatant was used for the ^1^H-NMR spectroscopy-based quantification of ethanol. Distilled water was used as negative control.

### ^1^H-NMR spectroscopy-based quantification of ethanol

^1^H-NMR analysis and quantification were performed as previously described with further modifications (Dorries and Lalk 2013). Samples were diluted 1:100 in pure water (HPLC-grade) and analyses were done in 5-mm-diameter glass tubes (103.5 mm long; Bruker Biospin, Rheinstetten, Germany). The Bruker AVANCE-NEO 600 NMR spectrometer equipped with a SampleJet autosampler and a 5-mm QCI cryo probe was operated by TOPSPIN v.4.0.6 software (Bruker Biospin). Quantification was done using AMIX software v.3.9.15 by integration and comparison of triplet peak of ethanol at 1.186 ppm to the ERETIC signal which was generated by using external calibration with the ERETIC quantification tool based on PULCON (Wider and Dreier 2006).

### Ethanol virus inactivation in cell culture

Hazara orthonairovirus (HAZV) was used as a model orthonairovirus for this study. HAZV was grown SW-13 cells with L-15 (Leibovitz) medium (Sigma-Aldrich, St. Louis, MO, USA) supplemented with 100 U/ml penicillin (Sigma-Aldrich), 0.1 mg/ml streptomycin (Sigma-Aldrich) and 2% fetal bovine serum. The virus was harvested 48 h post infection and the tissue culture infectious dose (TCID_50_) was calculated by the Spearman-Karber method. In order to create conditions similar to blood-fed infected ticks from the field and thus account for the possible influence of host blood, the virus-containing cell culture supernatant (10%) was mixed with bovine serum (80%) as well as bovine EDTA (10%). In this mixture, the virus was exposed to concentrations of 0–60% ethanol using a dilution series. The blood/virus mixture was eventually incubated under various temperature conditions (− 20 °C, 4 °C or room temperature) for four time periods (0, 3, 9, 24 h). Afterwards, the residual virus infectivity was determined by titration on SW-13 cells (6-well plates). Due to the cytotoxicity of ethanol, the virus cultivation at 6-well plates was conducted in an additional 1:20 dilution using the same media as described above. All samples were cultured in duplicate including positive and negative controls.

## Results

### Ethanol concentration in ticks

^1^H-NMR spectroscopic analysis revealed that the tick pools (1000 µl) contained 28.3–42.6 mg pure ethanol (Table 1). As a result, ethanol constituted 31–37.7% of the total weight of three ticks each per pool (89.9–121.5 mg) after 3 weeks of storage in ethanol. No ethanol was found in the negative control.

**Table 1:** Ethanol weight in relation to the total weight of the ticks after 3 weeks storage in 96% ethanol

Tick sex	Pool nr.	Ticks weight (mg)	Ethanol weight (mg)	Ratio (%)
Female	1	98.1	30.4	31.0
	2	81.9	30.9	37.7
	3	121.5	42.6	35.0
Male	4	108.5	36.2	33.3
	5	92.9	32.9	35.5
	6	107.3	38.0	35.5
	7	82.1	28.3	34.5

### Virus titration and cultivation under the influence of ethanol

**Fig. 1:**
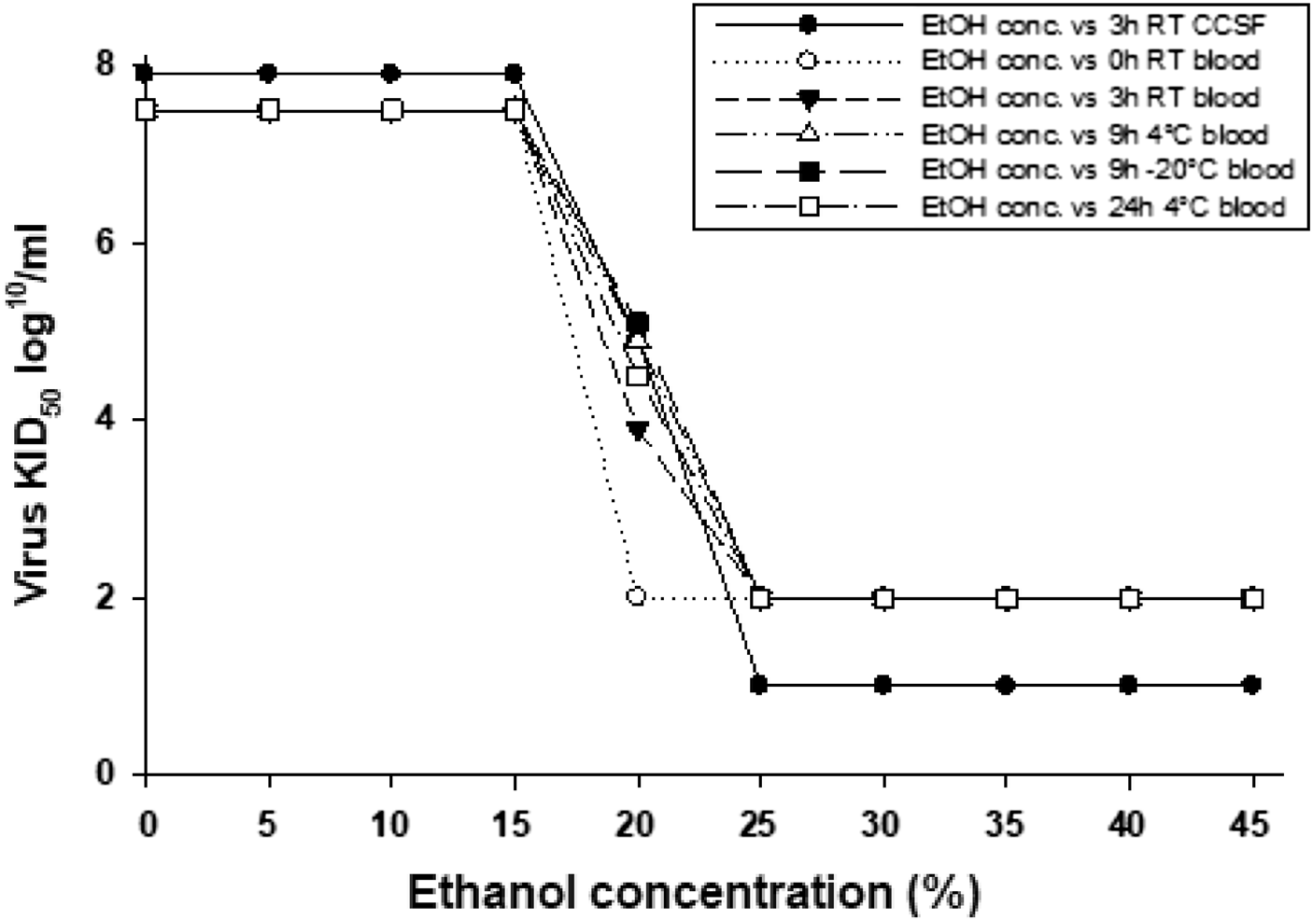
Titration results of Hazara orthonairovirus in various ethanol concentrations, at three temperatures (*RT* room temperature; *EtOH* conc., ethanol concentration; *CCSF* cell culture supernatant fluid). The figure was created with SigmaPlot v.11

The initial HAZV concentration in the cell culture supernatant was 10^7.8^/ml TCID_50_. Following ethanol exposure, a significant virus reduction started already at concentrations above 15% and a complete inactivation was observed with 20% ethanol (Fig. 1). It should be noted that ethanol concentrations above 25% were toxic to the cells. However, HAZV did not grow anymore in SW-13 cells after 9 h of exposure to an ethanol concentration of 25%.

## Discussion

In recent decades, the risk of emerging viral, bacterial and parasitic infectious diseases and their spread has risen considerably in response to globalization, climatic changes and increased human intervention in intact ecosystems. Studies have shown that tropical ticks and mosquito species are spreading continuously into previously non-endemic areas, thus increasing the risk of permanent introduction of (neglected) tropical diseases into these areas (Epstein 2001). Therefore, screening of tick vectors for pathogens in order to understand their occurrence and emergence has become an essential part of modern epidemiological research. In this context, a reliable inactivation of all potential infectious agents inside the ticks is crucial to ensure safe working conditions for the laboratory personnel in terms of biosafety / biosecurity.

The presented HAZV ethanol inactivation study results were generally in line with previously reported data (Hardestam et al. 2007) in which both CCHFV and HAZV infectivity was not detectable after remarkably short exposure times to 30 and 25% ethanol, respectively. Our results demonstrate that such concentrations are easily reached during a 3-week storage of unfed ticks in ethanol (96%). According to the quantitative ^1^H-NMR spectroscopic data, ethanol constitutes about one third of the mass of the examined tick pools (31.0–37.7%). The exact organ distribution of the alcohol inside the ticks or the actual dilution rate of the 96% ethanol cannot be calculated directly based on these data. However, due to the highly hydrophilic character of ethanol, it can be assumed that it will diffuse evenly into all aqueous compartments within the tick body, including the salivary glands and gastrointestinal tract. Nevertheless, the best proof of virus inactivation would be achieved by an in vivo inactivation study that should involve an experimental infection of ticks of varying feeding levels, the storage in ethanol and a re-isolation attempt of the virus.

### Conclusion

The obtained results indicate a suitably high ethanol diffusion into ticks to inactivate HAZV and CCHFV inside. Therefore, these data provide a proof of principle regarding the choice of adequate biosafety standards for laboratory work with ticks potentially harboring tick-borne pathogens.

## Data Availability

All data generated or analyzed during this study are included in this published article.
